# Cyt-C Mediated Mitochondrial Pathway Plays an Important Role in Oocyte Apoptosis in Ricefield Eel (*Monopterus albus*)

**DOI:** 10.3390/ijms231810555

**Published:** 2022-09-12

**Authors:** Zhi He, Qiqi Chen, Liang He, Jinxin Xiong, Kuo Gao, Bolin Lai, Li Zheng, Yong Pu, Yuanyuan Jiao, Zhijun Ma, Ziting Tang, Mingwang Zhang, Deying Yang, Taiming Yan

**Affiliations:** College of Animal Science and Technology, Sichuan Agricultural University, Chengdu 611130, China

**Keywords:** *Monopterus albus*, apoptosis, cytochrome-c (cyt-c), mitochondrial apoptotic pathway, sex change

## Abstract

Apoptosis plays a key role in the effective removal of excessive and defective germ cells, which is essential for sequential hermaphroditism and sex change in vertebrates. The ricefield eel, *Monopterus albus* is a protogynous hermaphroditic fish that undergoes a sequential sex change from female to male. Previous studies have demonstrated that apoptosis is involved in sex change in *M. albus*. However, the apoptotic signaling pathway is unclear. In the current study, we explored the underlying mechanism of apoptosis during gonadal development and focused on the role of the mitochondrial apoptosis signaling pathway in sex change in *M. albus*. Flow cytometry was performed to detect apoptosis in gonads at five sexual stages and ovary tissues exposed to hydrogen peroxide (H_2_O_2_) in vitro. Then the expression patterns of key genes and proteins in the mitochondrial pathway, death receptor pathway and endoplasmic reticulum (ER) pathway were examined. The results showed that the apoptosis rate was significantly increased in the early intersexual stage and then decreased with the natural sex change from female to male. Quantitative real-time PCR revealed that *bax*, *tnfr1*, and *calpain* were mainly expressed in the five stages. ELISA demonstrated that the relative content of cytochrome-c (cyt-c) in the mitochondrial pathway was significantly higher than that of caspase8 and caspase12, with a peak in the early intersexual stage, while the levels of caspase8 and caspase12 peaked in the late intersexual stage. Interestingly, the Pearson’s coefficient between cyt-c and the apoptosis rate was 0.705, which suggests that these factors are closely related during the gonadal development of *M. albus.* Furthermore, the cyt-c signal was found to be increased in the intersexual stage by immunohistochemistry. After incubation with H_2_O_2_, the mRNA expression of mitochondrial pathway molecules such as *bax*, *apaf-1*, and *caspase3* increased in ovary tissues. In conclusion, the present results suggest that the mitochondrial apoptotic pathway may play a more important role than the other apoptotic pathways in sex change in *M. albus.*

## 1. Introduction

Apoptosis, a type of programmed cell death [1], plays a crucial role in all stages of gonad development. Several studies have shown that apoptosis is involved in the elimination of germ cells from the ovaries [2,3] and testes [4,5], resulting in the production of high-quality gametes capable of fertilization. Apoptotic signals have been detected in oocytes of rainbow trout (*Oncorhynchus mykiss*) [6], redbelly tilapia (*Coptodon zillii*) [7], Nile tilapia (*Oreochromis niloticus*) [8], zebrafish (*Danio rerio*) [9], and *Prochilodus argenteus* [10] at different developmental stages, which suggests that apoptosis maintains an appropriate number of oocytes and eliminates unwanted cells. In addition, in the testes of *Cobitis taenia*, apoptosis occurs during the prespawning, spawning, and postspawning periods [4]. In sex-changing fish, apoptosis is essential for promoting sex reversal from female to male or male to female. In three-spot wrasse (*Halichoeres trimaculatus*), the degeneration of oocytes during sex change is controlled by apoptosis [11]. In zebrafish, the transition from ovary-like undifferentiated gonadal tissue to testes [12] and sex changes induced by external environmental factors are mediated by apoptosis [13]. These results indicate that apoptosis occurs during the physiological process of gonadal development in fishes.

The activation of cytochrome-c (cyt-c)/apoptotic protease activating factor 1 (apaf-1), Fas/tumor necrosis factor receptor 1 (Tnfr1), and caspases3/8/12 proteins have been shown to perform apoptotic physiological functions and are generally regarded as markers of apoptotic signaling pathways [3,14]. There are three classic pathways associated with apoptosis, including the mitochondrial pathway, death receptor pathway, and endoplasmic reticulum stress-induced apoptosis pathway all of which lead to activation of the caspases cascade [15,16,17,18,19,20]. The key to the mitochondrial pathway is the release of Cyt-c, which then interacts with Apaf-1 to form apoptosomes that activate downstream caspase9 to induce apoptosis [21,22]. The death receptor pathway is activated when Fas ligan and tumor necrosis factor α (TNFα) bind to the tumor necrosis factor receptor (FAS and TNFR1), and activate the downstream caspase8, to induce apoptosis. The endoplasmic reticulum (ER) pathway is triggered by the accumulation of misfolded and unfolded proteins, as well as interference with intracellular Ca^2+^ balance, thus allowing for downstream calpain and caspase12 activation that initiates apoptosis [23]. In zebrafish, three signaling pathways are involved in different apoptosis processes, including heart cell apoptosis through the mitochondrial pathway after isoliquiritigenin exposure [24], germ cell apoptosis through the ER pathway after microcystin-LR exposure [25], and the death receptor pathway mediates germ cell apoptosis during gonad differentiation [26]. Similarly, BDE-47 exposure induced apoptosis in rainbow trout gonadal cell line RTG-2 in the above three ways [14,27]. In addition, cadmium (Cd) induced neutrophil apoptosis via the mitochondrial and ER pathways in carp [28]. In conclusion, the mitochondrial pathway, death receptor pathway, and ER pathway are critical for the regulation of fish cell apoptosis.

The ricefeld eel (*Monopterus albus*), a protogynous hermaphroditic synbranchiform species, undergoes a sequential sex change from female to male, offering an interesting model for studying the mechanisms of sequential hermaphroditism in vertebrates [29]. Previous studies have demonstrated that a large number of oocytes degenerate and then disappear with the natural sex change from female to male. Furthermore, apoptotic molecular markers were detected, including *bcl2*, *p53*, *mdm2*, *siva1*, and *caspase3* [30,31,32,33], suggesting that apoptosis is involved in sex change in *Monopterus albus* However, the underlying apoptosis signaling pathways remain unclear. To explore whether three classical apoptotic pathways, including the mitochondrial pathway, the death receptor pathway, and the ER pathway, are involved in oocyte apoptosis during sex change, we measured the apoptosis rates of gonads at five different stages, analyzed the expression patterns of marker mRNAs and proteins in the three pathways, and incubated ovary tissue fragments with hydrogen peroxide (H_2_O_2_) in vitro. The results reveal apoptosis signaling mechanisms in the gonads that are related to sex change, enhancing our understanding of sequential hermaphroditism and sex change in *Monopterus albus* and other teleosts.

## 2. Results

### 2.1. Apoptosis Rates in Gonads at Different Developmental Stages

**Figure 1:**
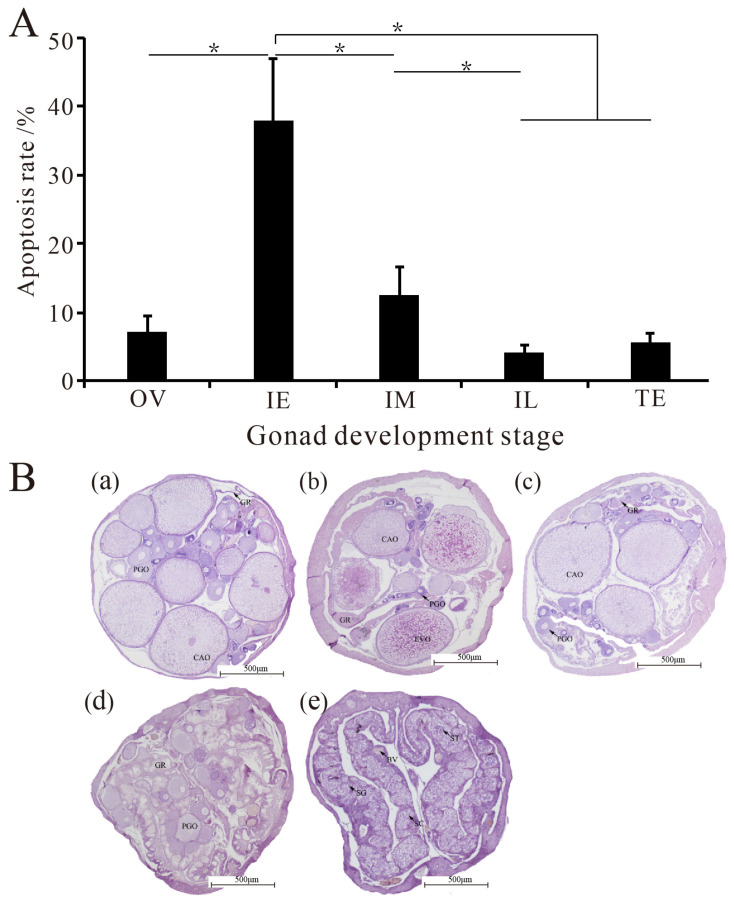
The apoptosis rates and histological changes in the gonad at different development stages of *Monopterus albus*. (**A**), Apoptosis rates in the five-development stage of gonad in *Monopterus albus*; (**B**), Identification of the five development stages of gonad in *Monopterus albus* based on histological changes. (**a**), OV, ovary; (**b**), IE, early intersexual stage gonad; (**c**), IM, middle intersexual stage gonad; (**d**), IL, late intersexual stage gonad; (**e**), TE, testis stage. CAO, cortical alveoli stage oocytes; PGO, primary growth stage oocytes; YO, yolk oocytes; GR, gonad ridge; SC, spermatocyte. The results are presented as the means ± SEMs. Asterisks (*) indicate significant differences between two groups in Figure 1A (*p* < 0.05).

Fluorescence-activated cell sorting (FACS) assays showed that various degrees of apoptosis occurred in the five different stages (Figure 1A). There was no significant difference in the apoptosis rate between the OV stage and the TE stage (*p* > 0.05), but the apoptosis rate in the IE stage was significantly higher than those in the other four stages (*p* < 0.05). In addition, with the completion of sex change (IE to IL), the apoptosis rate decreased significantly (*p* < 0.05). However, during the whole process of sex change, the apoptosis rate initially increased and then decreased gradually.

### 2.2. Relative Content of Apoptotic Proteins

**Figure 2:**
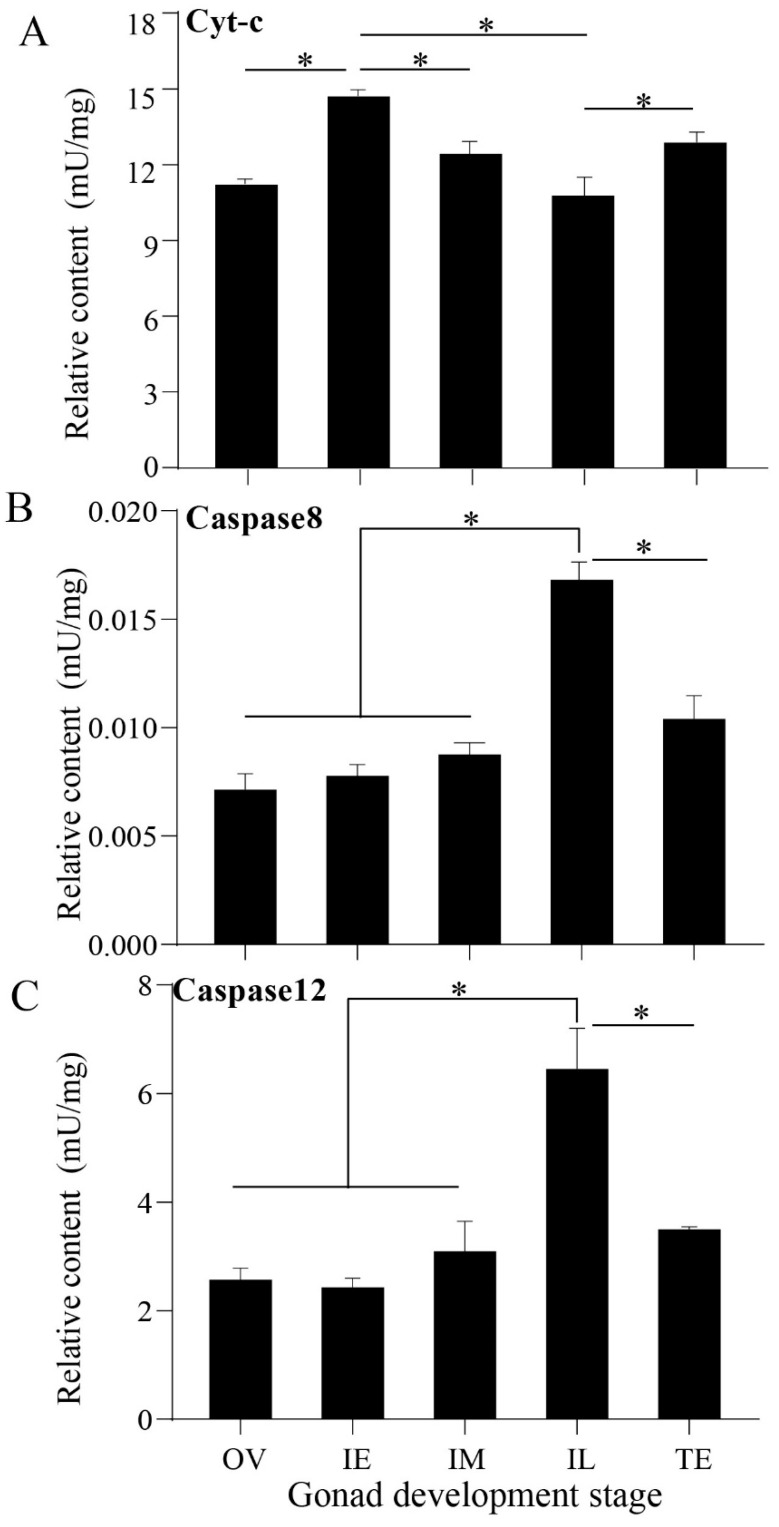
Expression patterns of apoptotic proteins related to the three signaling pathways during the gonadal development of *Monopterus albus*. (**A**), Expression patterns of cyt-c in the mitochondrial pathway; (**B**), Expression patterns of caspase-8 in the dead receptor pathway; (**C**), Expression patterns of caspase-12 in the ER pathway. OV, female stage ovary; IE, early intersexual stage ovary; IM, middle intersexual stage ovary; IL, late intersexual stage ovary; TE, testis. Asterisks (*) indicate significant differences between two groups (*p* < 0.05).

Cyt-c, caspase-8, and caspase-12 were detected in the five developmental stages (Figure 2). The protein expression of cyt-c was higher than that of than caspase-8 and caspase-12 (*p* < 0.05) and was highest in the IE stage (*p* < 0.05, Figure 2A). During the natural sex change of *Monopterus albus*, the protein levels of cyt-c increased significantly from the OV stage to the IE stage, and decreased significantly from the IE stage to the IL stage. Conversely, the expression of caspase-8 and caspase-12 was the highest in the IL stage (*p* < 0.05, Figure 2B,C). 

The Pearson’s coefficient between cyt-c and the apoptosis rate was 0.705, suggesting that these factors are closely related during the gonadal development of *Monopterus albus*, the details are provided in Table 1.

### 2.3. Expression Pattern of Apoptotic Genes in the Gonads at Different Developmental Stages 

**Figure 3:**
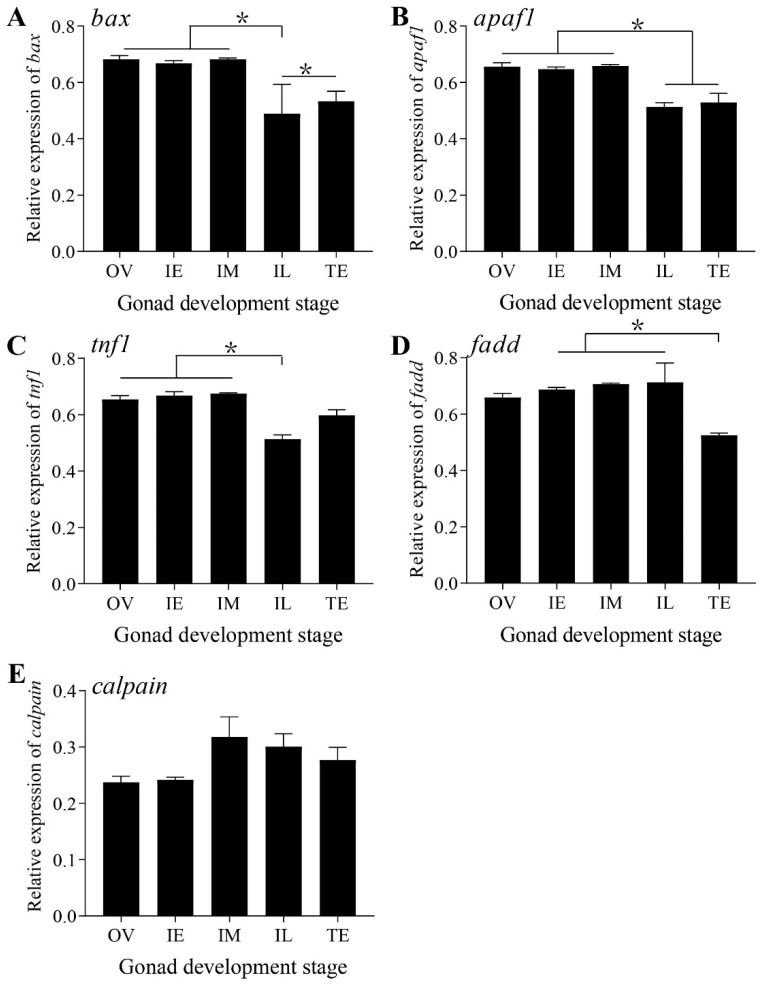
Expression patterns of apoptotic genes related to the three signaling pathways during the gonadal development of *Monopterus albus*. (**A**), Expression patterns of *bax* and *apaf-1* in the mitochondrial pathway. (**B**), Expression patterns of *fadd* and *tnfr1* in the dead receptor pathway. (**C**), Expression patterns of *calpain* in the ER pathway. OV, ovary; IE, early intersexual stage gonad; IM, middle intersexual stage gonad; IL, late intersexual stage gonad; TE, testis. The results are presented as the means ± SEMs. Asterisks (*) indicate significant differences between two groups (*p* < 0.05).

Marker genes of the three apoptosis signaling pathways were expressed in the five stages of *Monopterus albus* (Figure 3). There were no marked changes in the expression levels of *bax* and *apaf-1* in the mitochondrial pathways during the OV, IE, and IM stages (*p* > 0.05), but the levels decreased significantly in the IL stage (*p* < 0.05) and remained relatively stable until the TE stage (*p* > 0.05) (Figure 3A,B). In general, the expression of both in the OV stage was higher than that in the TE stage (Figure 3A,B). The expression patterns of *tnfr1* and *fadd* in the death receptor pathway were different: the expression pattern of *tnfr1* was similar to that of *bax*, while the expression pattern of *fadd* was similar to that of *calpain* in the ER pathway, which did not change significantly during the five stages (*p* < 0.05) (Figure 3C–E). However, the overall expression levels of the mitochondrial pathway genes were higher than those of the dead receptor pathway and ER pathway genes.

### 2.4. Immunolocalization of Cyt-C in the Gonads

**Figure 4:**
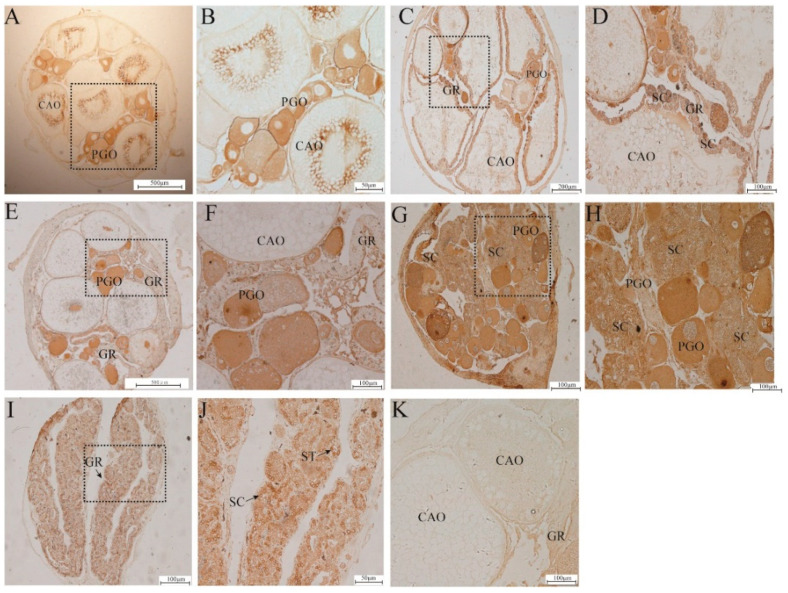
Immunohistochemical staining of cyt-c in the gonad tissues of *Monopterus albus* at different stages of development. (**A**,**B**), ovary; (**C**,**D**), early intersexual stage gonad; (**E**,**F**), middle intersexual stage gonad; (**G**,**H**), late intersexual stage gonad; (**I**,**J**), testis; (**K**), negative control. CAO, cortical alveoli oocyte; PGO, primary growth stage; YO, yolk oocytes; GC, granulosa cell; GR, gonadal ridge; SC, spermatocyte; ST, spermatid.

Cyt-c signals were detected in the five stages by immunohistochemical analysis. In the mid-vitellogenin stages, the cyt-c positive signal was detected in the cytoplasm of the primary growth-stage and cortical vesicle-stage oocytes but seemed to be stronger in the primary oocytes (Figure 4A,B). In the IE stage, both granulosa cells and theca cells on the ovarian mature oocyte membrane had cyt-c positive signals, and the cytoplasm of the mature yolk-stage, primary growth-stage and cortical vesicle-stage oocytes had a positive signal. Positive signals were also present in a small number of male germ cells above the gonadal ridge (Figure 4C,D). In the IM stage, the cytoplasm of oocytes in the primary growth stage and cortical vesicle phase was positive for the signal (Figure 4E,F). In the IL stage, almost all primary ovarian oocytes had positive cytoplasmic expression, and positive signals were observed on male germ cells above part of the gonad ridge (Figure 4G,H). In the testis, positive signals were strongly expressed in both spermatocytes and spermatids (Figure 4I,J). No positive signals were observed in the negative control when the primary antiserum was adsorbed with excessive antigen (Figure 4K).

### 2.5. Effect of H_2_O_2_ on Gonadal Apoptosis

**Figure 5:**
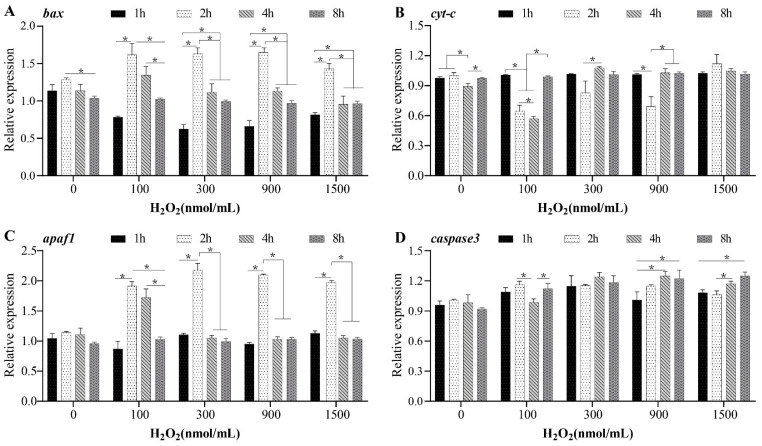
Expression patterns of mitochondrial pathway-related genes in *Monopterus albus* ovaries treated with H_2_O_2_ in vitro. (**A**), *Bax* expression patterns in ovaries after incubation with H_2_O_2_; (**B**), *Cyt-c* expression patterns in ovaries after incubation with H_2_O_2_; (**C**), *Apaf-1* expression patterns in ovaries after incubation with H_2_O_2_; (**D**), *Caspase3* expression patterns in ovaries after incubation with H_2_O_2_. Asterisks (*) indicate significant differences between two groups (*p* < 0.05).

The expression of *bax* in the 100, 300, 900, and 1500 nmol/mL treatment groups was significantly upregulated at 2 h (*p* < 0.5), and the expression level at 2 h was notably higher than the levels at 1 h, 2 h, and 8 h (*p* < 0.5) (Figure 5A). Similarly, the expression of *apaf-1* in the 100, 300, 900, and 1500 nmol/mL treatment groups increased at 2 h and then decreased significantly (*p* < 0.5) (Figure 5C). The expression level of *cyt-c* in the 100, 300, and 900 nmol/mL treatment groups significantly decreased at 1 h and then subsequently increased (*p* < 0.5) (Figure 5B). However, the expression level of *cyt-c* in the 1500 nmol/mL treatment groups did not change (*p* > 0.5) (Figure 5B). The expression of *caspase-3* under H_2_O_2_ treatment was greater than that in the control group, and the relative expression level of *caspase-3* in the 900 and 1500 nmol/mL treatment groups was significantly higher at 8 h than at 1 h (*p* < 0.5) (Figure 5D).

## 3. Discussion

Fish are the only vertebrates with functional hermaphroditism, and some undergo natural sex change during their life cycle. Protogyny (female-to-male sex change) is most pervasive in wrasses, such as bluehead (*Thalassoma bifasciatum*), temperate spotty (*Notolabrus celidotus*), and kyusen (*Parajulus poecilepterus*) wrasses, in which oocytes are removed through atresia during the sex change with apoptosis [34]. Histological observation of the gonads of the black porgy (*Acanthopagrus schlegeli*), a protandrous hermaphroditic fish, during the sex reversal process has shown that a large number of spermatocytes and spermatocytes disappear with apoptotic signals [35]. These results indicate that germ cells undergo apoptosis during sexual change. Similarly, *Monopterus albus* is also a protogynous hermaphroditic fish that undergoes sequential hermaphroditism from female to male via an intersex stage during its life cycle. The present study indicates that the apoptosis rates of germ cells, especially oocytes, increase significantly from the female stage to the IE stage and peak at the IE stage, suggesting that apoptosis plays a critical role in the sex change from female to male in *Monopterus albus.*

The process of mammalian gonadal apoptosis involves the death receptor pathway [3], the ER pathway [36] and the mitochondrial pathway [37]. Intracellular bax [38] and cyt-c [39]/apaf-1 expression are key regulators of the mitochondrial pathway. Cyt-c is released during the apoptosis of human oocytes [40]. The relative mRNA levels of *bax* are upregulated in mouse oocytes undergoing apoptosis [41]. However, the number of oocytes increases when *bax* is deleted [42]. Bax overexpression in rat oocytes shows typical morphological characteristics of apoptotic cells [43]. Therefore, bax- and cyt-c-mediated mitochondrial pathways play an important role in the apoptosis of female gonadal cells. In this study, the signature molecules of all three signaling pathways were detected, suggesting the coregulation of oocyte apoptosis during sex change. In particular, the mRNA expression levels of *bax* and *apaf-1* and the protein expression levels of cyt-c in the mitochondrial pathway were higher than those in the other two pathways, indicating that the mitochondrial pathway may play an important role in the sex change process of *Monopterus albus*.

It has been generally accepted that an increased level of cyt-c initiates apoptosis [44]. In *Gobiocypris rarus* spermatocytes treated with bisphenol A (BPA), mRNA expression of cyt-c is detectable, suggesting that the mitochondrial pathway is involved in apoptosis [45]. In the present study, the relative protein expression of cyt-c was significantly upregulated at the IE stage and then maintained at a relatively high level until the completion of sex change. Interestingly, cyt-c signals were enhanced in the cytoplasm of oocytes at the OV stage and detected predominantly in granulosa cells and theca cells in the IE stage, which suggests that cyt-c may play a crucial role in maintaining the number of germ cells and the apoptosis of oocytes at the IE stage.

H_2_O_2_ is a kind of reactive oxygen species (ROS) that can penetrate the cell membrane [46] and directly activate the mitochondrial PTP channel, releasing the mitochondrial protein cyt-c into the cytoplasm and then initiating the apoptotic cascade reaction [47]. H_2_O_2_ induces granulosa cell death, increases the expression of proapoptotic molecules such as bax and bak, and decreases the expression of antiapoptotic molecules such as bcl-2 and bcl-x [48]. After H_2_O_2_ incubation in vitro, all treatment groups showed an upwards trend, revealing that H_2_O_2_ induces an increase in caspase-3 expression. In this study, the expression levels of *apaf-1* and *bax* showed a trend of increasing and then decreasing expression, suggesting that H_2_O_2_-induced apoptosis may be more closely related to the mitochondrial pathway.

Apoptosis eliminates oocytes in the ovary and promotes sex change from female to male, suggesting that we could shorten the natural reversal cycle by inducing apoptosis of female germ cells and then obtain male *Monopterus albus* faster, which provides new insights for artificial sex control and improved mass production of larvae and juveniles in hatcheries.

## 4. Materials and Methods

### 4.1. Ethical Statement

All procedures and investigations were approved by the Animal Research and Ethics Committees of Sichuan Agricultural University and performed in accordance to the guidelines of the committee (Approval No.20190031).

### 4.2. Experimental Animals

Wild ricefield eels (*n* = 210, body length = 29.42 ± 5.13 cm and body weight = 48.53 ± 24.67 g) were purchased from a local market (Chengdu, Sichuan, China). The fish were maintained in the laboratory at a water temperature of 21.7 ± 2.5 °C under a photoperiod of 16 h light:8 h dark. Fish were decapitated after anesthesia with 0.02% tricaine buffer (80 μg/L) (Sigma, Saint Louis, MO, USA) and the gonads were collected and immediately stored in liquid nitrogen at −80 °C. The gonads were divided into two parts: the first part was fixed with Bouin’s solution for 24 h and then stored in 75% ethanol for determination of the gonadal developmental stage, and the second part was immediately stored in liquid nitrogen at −80 °C until RNA and protein extraction.

### 4.3. Developmental Stage Identification

The developmental stages of *Monopterus albus* gonads were determined using 5 μm thick histological sections and stained with haematoxylin–eosin [49]. According to a previous study [50], the sexual transition from female to male was classified into five phases: ovary (OV), early intersexual stage (IE), middle intersexual stage (IM), late intersexual stage (IL), and testis (TE).

### 4.4. Apoptosis Analysis

The gonads in different developmental stages were used to prepare tissue homogenate with a kit according to the manufacturer’s protocol (559763, BD, Franklin Lakes, NJ, USA). Then, a CytoFLEX flow cytometer (Beckman, Pasadena, CA, USA) was used to detect the apoptosis rate by flow cytometry with Annexin-V-PE and propidium iodide (PI) signals. Cell Quest software was used to analyze the results.

### 4.5. Determination of Relative Content of Apoptotic Proteins

The protein content of cyt-c, casepase12, and casepase8 in the gonads at the five developmental stages was determined by an ELISA kit (Shanghai Enzyme Biotechnology, Shanghai, China). In addition, a standard curve was prepared using bovine serum albumin (BSA), and the total soluble protein concentration was determined using a BCA total protein concentration assay kit. The relative content of apoptotic proteins in the sample was calculated as the ratio of the concentration of apoptotic protein to the concentration of total soluble protein in the homogenate.

### 4.6. RNA Isolation and Quantitative RT-PCR (qRT-PCR)

Total RNA was isolated from the gonads at different developmental stages using TRIzol reagent (Invitrogen, Chicago, IL, USA) and then reverse-transcribed into cDNA using a RevertAid First Strand cDNA Synthesis Kit (Thermo Scientific, Waltham, MA, USA) according to the manufacturer’s protocol. qRT–PCR was performed using a CFX Connect system (Bio-Rad, Chicago, IL, USA) in a final reaction volume of 10 μL comprising 5 μL of 2× SYBR Green Master Mix (TaKaRa, Dalian, China), 0.4 μL of each primer (10 μmol/L), 3.2 μL of nuclease-free water, and 1 μL of cDNA template. The cycling parameters were 95 °C for 5 min followed by 40 amplification cycles of 95 °C for 10 s, 59 °C for 15 s, and 72 °C for 20 s. The specificity of PCR amplification was confirmed by melting curve analysis, agarose gel electrophoresis, and sequencing of the PCR products.

The mRNA levels of *bax*, *apaf-1, caspase-3, tnfr1, calpain*, and *fadd* were normalized to the geometric mean expression levels of *ef1α* and *rpl 17* [51]. The details of all the primers for qRT-PCR used in this study are provided in Table 2.

### 4.7. Immunohistochemistry

Sections (5 μm thick) were deparaffinized, hydrated, and incubated in 3% H_2_O_2_ solution at room temperature for 30 min. After washing three times for 5 min each with PBS, the sections were incubated with 10% normal goat serum (Boster, Wuhan, China) for 30 min to block nonspecific reactions. Then, the sections were incubated for 1 h with a 1:100 dilution of anti-cyt-c (20R-1430, Fitzgerald) at room temperature, washed three times (5 min each) with PBS and incubated with the secondary antibody (HRP-conjugated goat anti-rabbit IgG) solution. After the final washes with PBS and incubation with the reagents of a DAB kit, the immunoreactivity of the sections was visualized. The sections were mounted, and the positive signals were examined with a digital imaging microscope system (Nikon, Eclipse Ti-S, Tokyo, Japan). Negative controls, including those in which the primary antibody was replaced with PBS, were used to confirm the specificity of the immunostaining.

### 4.8. H_2_O_2_ Incubation In Vitro

Female ricefield eels were purchased from Dazhong Breeding Limited Corporation (Jianyang, China). The ovaries were cleaned with cold PBS, minced to a size of approximately 1 mm^3^, randomly placed in 24-well plates, and cultured in 1 mL of Leibovitz L-15 medium (Gibco) containing penicillin (0.1 U/mL, Gibco) and streptomycin (0.1 mg/mL, Gibco) for preincubation for 4 h at 28 °C. After the medium was replaced with new L15 medium containing H_2_O_2_ at 100, 300, 900, and 1500 nmol/mL, the ovaries were incubated for 1, 2, 4, and 8 h and then collected. All samples were frozen in liquid nitrogen and stored at −80 °C until use. The mRNA expression levels of *caspase-3*, *apaf-1*, and *bax* were calculated from the qRT–PCR analysis as described above.

### 4.9. Statistical Analysis

All values are expressed as the mean ± SEM. The data were analyzed by one-way analysis of variance (ANOVA) followed by Tukey’s multiple comparisons test using SPSS 21.0 (IBM, Armonk, NY, USA). Finally, Pearson’s product-moment was used for correlation analysis. The significance was set at *p* < 0.05.

## 5. Conclusions

The present study is the first to report that the mitochondrial pathway, ER pathway and death receptor pathway are involved in oocyte apoptosis during sex change in the ricefield eel *Monopterus albus*. The results showed that apoptosis occurred throughout gonadal development, with the highest apoptosis rate in the IE stage. The expression levels of marker genes and proteins of the mitochondrial pathway were higher than those of the death receptor pathway and ER pathway. In addition, the Pearson’s coefficient between cyt-c and the apoptosis rate was 0.705. The cyt-c positive signals were stronger in the intersex stage and mitochondrial pathway gene mRNA expression levels increased after H_2_O_2_ incubation in vitro, suggesting that the mitochondrial pathway may play a more important role in oocyte apoptosis during sex change in *Monopterus albus*.

## Figures and Tables

**Table 1:** Correlation analysis between the apoptosis rate and apoptotic proteins at the IE stage.

		Caspase-12	Cyt-C	Caspase-8
Apoptosis rate	Pearson correlation	−0.377	0.705 **	−0.386
	Sig. (2-tailed)	0.063	0.001	0.056
	N	25	25	25

Note: “**” significance at the 0.01 level. “N” indicates the number of samples.

**Table 2:** Primers used for real-time quantitative PCR analysis.

Primer	Sequence (5′-3′)
*bax* F	CTTTGCCTGTCGGCTTGTCA
*bax* R	ATACCCTCCCAGCCACCTTG
*apaf-1* F	TAAGAACCCCTCTGATGGCTCC
*apaf-1* R	ATTCCAAACACAGTGACCCAGC
*caspase-3* F	GCGGACTTCCTCTATGC
*caspase-3* R	CAAGGTGGCAGCAGAGT
*tnfr1* F	TCCACCTGGGGACTACGCTAC
*tnfr1* R	ACTGTCCAAGAGGGCAAGGC
*calpain* F	TGAAGGGCGGAAACACCACC
*calpain* R	CTCAAAGCGAGCGGGAACCA
*fadd* F	GCCGACACAACGGAGTATCT
*fadd* R	TTACCTCTGTGGCGATGTTC
*ef1α* F	CGCTGCTGTTTCCTTCGTCC
*ef1α* R	TTGCGTTCAATCTTCCATCCC
*rpl 17* F	GTTGTAGCGACGGAAAGGGAC
*rpl 17* R	GACTAAATCATGCAAGTCGAGGG

F: sense primer; R: antisense primer.

## Data Availability

Not applicable.

## References

[1] Bosco L., Ruvolo G., Morici G., Manno M., Cittadini E., Roccheri M.C. (2005). Apoptosis in human unfertilized oocytes after intracytoplasmic sperm injection. Fertil. Steril..

[2] Yadav P.K., Tiwari M., Gupta A., Sharma A., Prasad S., Pandey A.N., Chaube S.K. (2018). Germ cell depletion from mammalian ovary: Possible involvement of apoptosis and autophagy. J. Biomed. Sci..

[3] Tiwari M., Prasad S., Tripathi A., Pandey A.N., Ali I., Singh A.K., Shrivastav T.G., Chaube S.K. (2015). Apoptosis in mammalian oocytes: A review. Apoptosis.

[4] Jablonska O., Juchno D., Leska A., Kowalewska K. (2020). The variable presence of apoptosis in the testes of diploid and sterile allotetraploid Cobitis (Teleostei, Cobitidae) males during reproductive cycle. J. Exp. Biol..

[5] Ribeiro Y.M., de Matos S.A., Domingos F.F.T., Dos Santos H.B., Bicalho A., Vieira C., Bazzoli N., Rizzo E. (2017). Germ cell proliferation and apoptosis during testicular regression in a seasonal breeding fish kept in captivity. Tissue Cell.

[6] Wood A.W., Kraak G.V.D. (2002). Inhibition of apoptosis in vitellogenic ovarian follicles of rainbow trout (Oncorhynchus mykiss) by salmon gonadotropin, epidermal growth factor, and 17β-estradiol. Mol. Reprod. Dev..

[7] Mokhtar D.M., Hussein M. (2020). Microanalysis of Fish Ovarian Follicular Atresia: A Possible Synergic Action of Somatic and Immune Cells. Microsc. Microanal..

[8] Sales C.F., Melo R.M.C., Pinheiro A.P.B., Luz R.K., Rizzo E. (2019). Autophagy and Cathepsin D mediated apoptosis contributing to ovarian follicular atresia in the Nile tilapia. Mol. Reprod. Dev..

[9] Rodríguez-Marí A., Cañestro C., Bremiller R.A., Nguyen-Johnson A., Asakawa K., Kawakami K., Postlethwait J.H. (2010). Sex reversal in zebrafish fancl mutants is caused by Tp53-mediated germ cell apoptosis. PLoS Genet..

[10] Thomé R., Domingos F., Santos H.B., Martinelli P.M., Sato Y., Rizzo E., Bazzoli N. (2012). Apoptosis, cell proliferation and vitellogenesis during the folliculogenesis and follicular growth in teleost fish. Tissue Cell.

[11] Ryo N., Ryo H., Ryosuke M., Yasuhisa K., Masaru N. (2013). Survival of ovarian somatic cells during sex change in the protogynous wrasse, *Halichoeres trimaculatus*. Fish Physiol. Biochem..

[12] Uchida D., Yamashita M., Kitano T., Iguchi T. (2002). Oocyte apoptosis during the transition from ovary-like tissue to testes during sex differentiation of juvenile zebrafish. J. Exp. Biol..

[13] Uchida D., Yamashita M., Kitano T., Iguchi T. (2004). An aromatase inhibitor or high water temperature induce oocyte apoptosis and depletion of P450 aromatase activity in the gonads of genetic female zebrafish during sex-reversal. Comp. Biochem. Physiol..

[14] Zhou Z., Zhou B., Chen H., Tang X., Wang Y. (2019). Reactive oxygen species (ROS) and the calcium-(Ca^2+^) mediated extrinsic and intrinsic pathways underlying BDE-47-induced apoptosis in rainbow trout (*Oncorhynchus mykiss*) gonadal cells. Sci. Total Environt..

[15] Bridgham J., Wilder J., Hollocher H., Johnson A. (2003). All in the family: Evolutionary and functional relationships among death receptors. Cell Death Differ..

[16] Redza-Dutordoir M., Averill-Bates D.A. (2018). Activation of apoptosis signaling pathways by reactive oxygen species. Biochim. Biophys. Acta-Mol. Cell Res..

[17] Zhao S., Yuan C., Tuo X., Zhou C., Zhao Q., Shen T. (2020). MCLR induces dysregulation of calcium homeostasis and endoplasmic reticulum stress resulting in apoptosis in Sertoli cells. Chemosphere.

[18] Giamogante F., Poggio E., Barazzuol L., Covallero A., Calì T. (2021). Apoptotic signals at the endoplasmic reticulum-mitochondria interface. Adv. Protein Chem. Struct. Biol..

[19] Kawamura K., Fukuda J., Kodama H., Kumagai J., Kumagai A., Tanaka T. (2001). Expression of Fas and Fas ligand mRNA in rat and human preimplantation embryos. Mol. Hum. Reprod..

[20] Fu X., Cui J., Meng X., Jiang P., Zheng Q., Zhao W., Chen X. (2021). Endoplasmic reticulum stress, cell death and tumor: Association between endoplasmic reticulum stress and the apoptosis pathway in tumors (Review). Oncol. Rep..

[21] Shakeri R., Kheirollahi A., Davoodi J. (2017). Apaf-1: Regulation and function in cell death. Biochimie (Paris).

[22] Wang H., Zhu J., Jiang L., Shan B., Xiao P., Ai J., Li N., Qi F., Niu S. (2020). Mechanism of Heshouwuyin inhibiting the Cyt c/Apaf-1/Caspase-9/Caspase-3 pathway in spermatogenic cell apoptosis. BMC Complement. Altern. Med..

[23] Zuo S., Kong D., Wang C., Liu J., Wang Y., Wan Q., Yan S., Zhang J., Tang J., Zhang Q. (2018). CRTH2 promotes endoplasmic reticulum stress-induced cardiomyocyte apoptosis through m-calpain. EMBO Mol. Med..

[24] Song Z., Zhang Y., Zhang H., Rajendran R.S., Wang R., Hsiao C.-D., Li J., Xia Q., Liu K. (2020). Isoliquiritigenin triggers developmental toxicity and oxidative stress-mediated apoptosis in zebrafish embryos/larvae via Nrf2-HO1/JNK-ERK/mitochondrion pathway. Chemosphere.

[25] Zhan C., Liu W., Zhang F., Zhang X. (2020). Microcystin-LR triggers different endoplasmic reticulum stress pathways in the liver, ovary, and offspring of zebrafish (*Danio rerio*). J. Hazrd. Mater..

[26] Luzio A., Matos M., Santos D., Fontaínhas-Fernandes A.A., Monteiro S.M., Coimbra A.M. (2016). Disruption of apoptosis pathways involved in zebrafish gonad differentiation by 17α-ethinylestradiol and fadrozole exposures. Aquat. Toxicol..

[27] Zhou Z., Chen H., Li Y., Liu Q., Lu K., Zhu X., Wang Y. (2022). Transcriptome and biochemical analyses of rainbow trout (*Oncorhynchus mykiss*) RTG-2 gonadal cells in response to BDE-47 stress indicates effects on cell proliferation. Aquat. Toxicol..

[28] Sun J., Wang S., Cao Y., Wang S., Li S. (2020). Cadmium exposure induces apoptosis, inflammation and immunosuppression through CYPs activation and antioxidant dysfunction in common carp neutrophils. Fish Shellfish. Immunol.

[29] Liu C.K. (1944). Rudimentary hermaphroditism in the symbranchoid eel, Monopterus javanensis. Sinensia.

[30] He Z., Deng F., Ma Z., Zhang Q., He J., Ye L., Chen H., Yang D., He L., Luo J. (2021). Molecular characterization, expression, and H2O2 induction of p53 and mdm2 in the ricefield eel. Monopterus Albus. Aquac. Rep..

[31] He Z., Deng F., Ma Z., Zhang Q., He J., Ye L., Chen H., Yang D., He L., Luo J. (2021). Molecular characterization, expression, and apoptosis regulation of siva1 in protogynous hermaphrodite fish ricefield eel (Monopterus albus). Fish Physiol. Biochem..

[32] He Z., He Z., He L., Ruan H., Li S., Yan T. (2019). Expression and localization of Caspase-3 in *Monopterus albus* gonad during natural sex reversal (in Chinese). Freshwater Fish..

[33] Shi Y., Zhong L., Chen K., Fan Y., Xie K., Zhang J. (2022). Sanguinarine attenuates hydrogen peroxide-induced toxicity in liver of *Monopterus albus*: Role of oxidative stress, inflammation and apoptosis. Fish Shellfish Immun..

[34] Thomas J.T., Todd E.V., Muncaster S., Lokman P.M., Damsteegt E.L., Liu H., Soyano K., Gléonnec F., Lamm M.S., Godwin J.R. (2019). Conservation and diversity in expression of candidate genes regulating socially-induced female-male sex change in wrasses. PeerJ.

[35] Wu G.-C., Chang C.-F. (2012). Oocytes Survive in the Testis By Altering the Soma Fate from Male to Female in the Protandrous Black Porgy, *Acanthopagrus schlegeli*. Biol. Reprod..

[36] Tosti E. (2006). Calcium ion currents mediating oocyte maturation events. Reprod. Biol. Endocrinol..

[37] Gabriel B., Sureaua F., Casselyn M., Teissié J., Petit P.X. (2003). Retroactive pathway involving mitochondria in electroloaded cytochrome c-induced apoptosis: Protective properties of Bcl-2 and Bcl-XL. Exp. Cell Res..

[38] Stallock J., Molyneaux K., Schaible K., Knudson C.M., Wylie C. (2003). The pro-apoptotic gene Bax is required for the death of ectopic primordial germ cells during their migration in the mouse embryo. Clin. Exp. Obstet. Gynecol..

[39] Rucker E.I., Dierisseau P., Wagner K.U., Garrett L., Hennighausen L. (2000). Bcl-x and Bax Regulate Mouse Primordial Germ Cell Survival and Apoptosis during Embryogenesis. Mol. Endocrinol..

[40] Zhang X., Li X.-H., Ma X., Wang Z.-H., Lu S., Guo Y.-L. (2006). Redox-Induced Apoptosis of Human Oocytes in Resting Follicles In Vitro. J. Soc. Gynecol. Investig..

[41] Felici M.D., Carlo A.D., Pesce M., Iona S., Farrace M.G., Piacentini M. (1999). Bcl-2 and Bax regulation of apoptosis in germ cells during prenatal oogenesis in the mouse embryo. Cell Death Differ..

[42] Greenfeld C.R., Pepling M.E., Babus J.K., Furth P.A., Flaws J.A. (2007). BAX regulates follicular endowment in mice. Reproduction.

[43] Chaube S.K., Prasad P.V., Thakur S.C., Shrivastav T.G. (2005). Hydrogen peroxide modulates meiotic cell cycle and induces morphological features characteristic of apoptosis in rat oocytes cultured in vitro. Apoptosis.

[44] Song X.F., Tian H., Zhang P., Zhang Z.X. (2017). Expression of Cyt-c-Mediated Mitochondrial Apoptosis-Related Proteins in Rat Renal Proximal Tubules during Development. Nephron.

[45] Zhang Y., Cheng M., Wu L., Zhang G., Wang Z. (2016). Bisphenol A induces spermatocyte apoptosis in rare minnow Gobiocypris rarus. Aquat. Toxicol. (Amst. Neth.).

[46] Pasquali M.A.B., Gelain D.P., Zanotto-Filho A., de Souza L.F., de Oliveira R.B., Klamt F., Moreira J.C.F. (2008). Retinol and retinoic acid modulate catalase activity in Sertoli cells by distinct and gene expression-independent mechanisms. Toxicol. In Vitro.

[47] Wang X., Yuan X., Sun Y., Wu J., Zhang J. (2009). Effect of H2O2 on apoptosis of sertoli cell. Chin. J. Vet..

[48] Yang H., Yan X., Yang D., Ren D. (2017). Oxidative stress-induced apoptosis in granulosa cells involves JNK, p53 and Puma. Oncotarget.

[49] Nowosad J., Kucharczyk D., Luczyńska J., Targońska K., Czarkowski T.K., Bilas M. (2014). Changes in European eel ovary development and body and ovary chemistry during stimulated maturation under controlled conditions: Preliminary data. Aquac Int..

[50] He Z., Wu Y., Xie J., Wang T., Zhang L., Zhang W. (2012). Growth differentiation factor 9 (Gdf9) was localized in the female as well as male germ cells in a protogynous hermaphroditic teleost fish, ricefield eel *Monopterus albus*. Gen. Comp. Endocrinol..

[51] Hu Q., Guo W., Gao Y., Tang R., Li D. (2014). Reference gene selection for real-time RT-PCR normalization in rice field eel (*Monopterus albus*) during gonad development. Fish Physiol. Biochem..

